# Occurrence of a RAGE-Mediated Inflammatory Response in Human Fetal Membranes

**DOI:** 10.3389/fphys.2020.00581

**Published:** 2020-06-25

**Authors:** Héléna Choltus, Marilyne Lavergne, Corinne Belville, Denis Gallot, Régine Minet-Quinard, Julie Durif, Loïc Blanchon, Vincent Sapin

**Affiliations:** ^1^CNRS, INSERM, GReD, Université Clermont Auvergne, Clermont-Ferrand, France; ^2^CHU de Clermont-Ferrand, Obstetrics and Gynecology Department, Clermont-Ferrand, France; ^3^CHU de Clermont-Ferrand, Biochemistry and Molecular Genetic Department, Clermont-Ferrand, France

**Keywords:** fetal membranes, RAGE, alarmins, sterile inflammation, rupture of fetal membranes

## Abstract

**Context:**

Sterile inflammation has been shown to play a key role in the rupture of the fetal membranes (FMs). Moreover, an early and exacerbated runaway inflammation can evolve into a preterm premature rupture of membranes and lead to potential preterm birth. In this context, we investigated the receptor for advanced glycation end products (RAGE), an axis implied in physiological sterile inflammation, in conjunction with two major ligands: AGEs and High-Mobility Group Box 1 (HMGB1). Our first objective was to determine the spatiotemporal expression profiles of the different actors of the RAGE-signaling axis in human FMs, including its intracellular adaptors Diaphanous-1 and Myd88. Our second goal was to evaluate the functionality of RAGE signaling in terms of FMs inflammation.

**Methods:**

The presence of the actors (RAGE, HMGB1, Myd88, and Diaphanous-1) at the mRNA level was investigated by reverse transcription quantitative polymerase chain reaction (RT-qPCR) in the human amnion and choriodecidua at the three trimesters and at term. Measurements were conducted at two distinct zones: the zone of intact morphology (ZIM) and the zone of altered morphology (ZAM). Then, proteins were quantified using Western blot analysis, and their localization was evaluated by immunofluorescence in term tissues. In addition, pro-inflammatory cytokine secretion was quantified using a Multiplex assay after the treatment of amnion and choriodecidua explants with two RAGE ligands (AGEs and HMGB1) in the absence or presence of a RAGE inhibitor (SAGEs).

**Results:**

The FMs expressed the RAGE-signaling actors throughout pregnancy. At term, RNA and protein overexpression of the RAGE, HMGB1, and Diaphanous-1 were found in the amnion when compared to the choriodecidua, and the RAGE was overexpressed in the ZAM when compared to the ZIM. The two RAGE ligands (AGEs and HMGB1) induced differential cytokine production (IL1β and TNFα) in the amnion and choriodecidua.

**Conclusion:**

Considered together, these results indicate that RAGE signaling is present and functional in human FMs. Our work opens the way to a better understanding of FMs weakening dependent on a RAGE-based sterile inflammation.

## Introduction

Fetal membranes are an essential actor in human parturition; if they do not achieve their missions, the childbirth can be impacted (Naeye and Peters, 1980; Romero et al., 2006; Menon, 2016; Menon and Richardson, 2017). These fetal tissues consist of two layers: the amnion, which is the innermost layer directly in contact with the amniotic fluid (AF), and the chorion, which adheres to the maternal decidua. This 9-month organ participates in the correct development of the fetus by providing AF homeostasis as well as physical and microbial barriers during pregnancy; however, they also play a role in parturition by their programmed rupture at term (after 37 weeks gestation) (Buhimschi et al., 2004; Moore et al., 2006; King et al., 2007; Prat et al., 2012). In this way, FMs undergo progressive weakening leading to this physiologic rupture of membranes (ROM) thanks to several mechanisms, such as apoptosis, senescence, or inflammation (Parry and Strauss, 1998; Menon et al., 2019).

Recently, an increasing number of studies have shown the implication of one key phenomenon in the FMs weakening: sterile inflammation (Girard et al., 2014; Romero et al., 2014, 2015). This concept is dependent on specific molecules called alarmins or “damage-associated molecular patterns” (DAMPs), which are released and recognized by pattern recognition receptors (PRRs) leading to a microbial-free inflammatory response or a “sterile” inflammation. Examples of DAMPs include high-mobility group box 1 (HMGB1) protein, the S100 protein family, uric acid, cell-free DNA, and advanced glycation end-products (AGEs), and examples of PRRs include toll-like receptors, scavenger receptors, NOD-like receptors, and the receptor for AGEs (Taglauer et al., 2014; Nadeau-Vallée et al., 2016; Brien et al., 2019). It has been determined that AF contains many of these alarmins, which induce pro-inflammatory cytokine release by activating various cellular pathways (Holmlund et al., 2007; Jakobsen et al., 2012; Bredeson et al., 2014; Menon and Moore, 2020). Lappas and colleagues demonstrated an induction of cytokine release (IL1β, IL6, IL8, TNFα) by FMs in response to AGEs (Lappas et al., 2007).

However, it still remains unclear how this phenomenon works exactly or which receptor translates this inflammatory signaling to the FMs to prepare for a successful ROM that does not occur before 37 weeks. In the case of early activation, preterm prelabor rupture of the membranes (pPROM) can occur. pPROM affects 3–4% of all pregnancies and leads to 30–40% of all preterm births. Yearly, there are about 15 million cases of preterm birth worldwide. It is important to note that this problem is associated with the rise of perinatal mortality, morbidity, and developmental troubles (Schreiber and Benedetti, 1980; Silverman and Wojtowycz, 1998; Fujimoto et al., 2002; England et al., 2013; Lorthe, 2018; Bouvier et al., 2019; Shiqiao et al., 2019). Thus, it is essential to better understand the ROM to improve pPROM diagnostics and clinical care.

In this study, we decided to investigate the implication of one actor: RAGE (Neeper et al., 1992; Brett et al., 1993). Originally discovered in 1992 as a new member of the immunoglobulin superfamily of receptors, the RAGE is a 55 kDa cell surface receptor that interacts with several ligands (including AGEs and HMGB1) implicated in the pathogenesis of many inflammatory diseases (Kierdorf and Fritz, 2013; Ray et al., 2016; Hudson and Lippman, 2018). Indeed, the RAGE is known to activate pro-inflammatory pathways and the release of cytokines and has been described as participating in the weakening of FMs (Rzepka et al., 2015). Plus, lower concentrations of a soluble RAGE, a competitive RAGE isoform lacking the intracellular domain, has been discovered in the maternal serum of patients suffering from pPROM (Hájek et al., 2008). Furthermore, even if the expression of the RAGE has been outlined in the placental sphere, little is known about the RAGE in FMs and even less on the action and physiopathology of the RAGE in terms of the ROM (Yan et al., 2018). Plus, it is well known that RAGE signaling activity relies on its interaction with intracellular adaptors proteins such as Diaphanous-1, Myd88, and TIR adaptor protein (TIRAP) (Hudson et al., 2008; Sakaguchi et al., 2011). Thus, this study intends to provide more information about the RAGE axis actors in the FMs and determine if a RAGE-dependent inflammatory response can specifically occur in the amnion or choriodecidua when exposed to alarmins such as HMGB1 and AGEs.

## Materials and Methods

### Chemicals

HMGB1 (SRP6265, 10 μg/mL in phosphate-buffered saline 1X) was purchased from Sigma-Aldrich (Saint-Quentin-Fallavier, France) and AGE-bovine serum albumin (10 mg/mL, ab51995) from Abcam (Paris, France). Semi-synthetic glycosaminoglycan ethers (SAGEs) (GM-1111, 10 mg/mL in water), used for the RAGE inhibition, were kindly gifted by GlycoMira Therapeutics (Salt Lake City, UT, United States) (Zhang et al., 2011). Cell culture medium and antibiotics (streptomycin, penicillin, amphotericin B) were obtained from Fisher Scientific (Illkirch-Graffenstaden, France). Fetal bovine serum (FBS) was purchased from Eurobio Scientific (Les Ulis, France). Collagen I was obtained from Stemcell Technologies (Grenoble, France). Superscript IV first-strand-synthesis system, Taq DNA polymerase recombinant (10342020), and Pierce BCA protein assay kit (23225) were obtained from Fisher Scientific.

### Tissue Collection

Full-term FMs were collected from non-smoking women with healthy pregnancies from vaginal or scheduled cesarean deliveries (breech presentation, scarred wombs) (Centre Hospitalier Universitaire Estaing, Clermont-Ferrand, France) after obtaining informed consent. Gestational ages were 39.08 ± 0.11 weeks, mean maternal ages were 35.30 ± 0.94 years, and maternal body mass index (BMI) was 26.64 ± 6.65. The selected FMs were collected from singleton pregnant women who had no underlying diseases and no gestational diabetes or clinical chorioamnionitis (defined by maternal fever, uterine tenderness, and/or purulent amniotic fluid). The research protocol was approved by the institutional regional ethics committee (DC-2008-558). The amnion was dissociated from the choriodecidua. The zone of altered morphology (ZAM, with the thread) and the zone of intact morphology (ZIM, away from the thread) were also distinguished. Indeed a suture sewn placed onto the FMs (from cesarean deliveries) in front of the cervix by the midwife allowed us to identify ZAM; then, a 4-cm-diameter circle was cut and considered as ZAM, and explants localized places away from circle boundary were considered as ZIM.

Concerning samples used for RAGE axis actor exploration throughout the pregnancy, first-trimester membranes (*N* = 3) were obtained following aspiration after voluntary termination of pregnancy. Second-trimester membranes were harvested after medical termination of pregnancy (*N* = 3). Eligible cases corresponded to lethal fetal anomalies that had no impact on the FMs (e.g., severe cardiac anomalies or brain damage). Then, preterm third-trimester membranes (*N* = 3) were collected from pregnancies after cesarean births. The amnion was dissociated from the choriodecidua except for trimester 1 samples.

### Tissue Culture

Explants (dissociated) of the amnion and choriodecidua were cultivated (5% CO_2_, 95% humidified air, 37°C) in Dulbecco’s modified eagle medium/nutrient mixture F-12 (DMEM-F12- GlutaMAX) supplemented with 10% FBS, 100 μg/ml of streptomycin, 100 U/ml of ampicillin, and 25 μg/ml amphotericin B. Explants were 2 cm^2^ in size, obtained 2 cm away from the pre-placental edge and prepared by dissection. Tissue fragments were transferred (in duplicate) to 24-well culture plates and incubated in cell media at 37°C for 1 h before treatment.

### Tissue Explant Treatment

Explants were treated with AGEs (150, 250, and 500 μg/ml) or HMGB1 (100, 200, and 300 ng/ml) in the absence or presence of SAGEs (500 μg/ml) for 18 h (cell medium collection for cytokine release assay). In addition, an internal control was performed by treating explants with a combination of lipopolysaccharide (LPS) (10 μg/ml) and TNFα (100 ng/ml) to validate inflammatory reactivity of FMs samples used. FMs were validated when there was a release response of at least one cytokine.

### RT-PCR and Quantitative RT-PCR

After the disruption step with Precellys homogenizer (Bertin Technologies, Montigny-le-Bretonneux, France) using ceramic beads (KT03961, Ozyme, Saint-Cyr-l’École, France), total RNAs were extracted from human amnion or choriodecidua using RNAzol^®^ RT (RN190, Molecular Research Center, Cincinnati, OH, United States). The reverse transcription was made from 1 μg of RNA using a Superscript IV first-strand-synthesis system for reverse transcription polymerase chain reaction (RT-PCR). PCR experiments were performed using specific oligonucleotides (Table 1). Results were analyzed on a 2% agarose gel and verified by DNA sequencing. RAGE, HMGB1, Myd88, and Diaphanous-1 expression was assessed by quantitative RT-PCR (RT-qPCR) performed using LightCycler^®^ 480 SYBR Green I Master (Roche, Meylan, France). Transcript quantification was performed twice on at least four independent experiments. Results were normalized to the geometric mean of the human housekeeping genes RPL0 (36b4) and RPS17 (acidic ribosomal phosphoprotein P0 and ribosomal protein S17, respectively) as recommended by the MIQE guidelines (Bustin et al., 2009).

**TABLE 1:** Forward and reverse primer sequences used for RT-PCR and RT-qPCR amplification of human genes.

Gene	Sequence 5′-3′ (F: forward, R: reverse)	Product size (bp)	Hybridation temperature (°C)
*hsRAGE*	F:TGTGCTGATCCTCCCTGAGA R: CGAGGAGGGGCCAACTGCA	139	61
*hsRPL0/36B4*	F: AGGCTTTAGGTATCACCACT R: GCAGAGTTTCCTCTGTGATA	219	61
*hsRSP17*	F: TGCGAGGAGATCGCCATTATC R: AAGGCTGAGACCTCAGGAAC	169	61
*hsMyd88*	F: GCAGGAGGAGGCTGAGAAGC R: CGGATCATCTCCTGCACAAACT	167	63
*hsDia-1*	F: AGAGCCACACTTCCTTTCCATC R: TCAATCTCAATCTGGAGGTGCC	167	61
*hsHMGBI*	F: ACCTATATCCCTCCCAAAGGG R: TTTTTGGGCGATACTCAGAGCA	109	61

### Western Blot Analysis

After the preliminary tissue homogenization previously described, total proteins were extracted from human amnion and choriodecidua (total, ZIM, or ZAM) with a plasma membrane protein extraction kit (BioVision, Lyon, France), and protein sample concentrations were measured using a Pierce BCA protein assay kit. For Western blot analysis, proteins were resolved on a 4–15% Mini-PROTEAN^®^ TGX Stain-Free^TM^ Precast Gel (Bio-Rad, Marnes-la-Coquette, France) to perform total protein normalization (Gilda and Gomes, 2013). Before transfer, stain-free imaging was completed. This technology utilizes a proprietary trihalo compound to enhance natural protein fluorescence by covalently binding to tryptophan residues with a brief UV activation (Bio-Rad). Then, the transfer was performed on nitrocellulose membrane (Bio-Rad) and saturated over 1 h 30 min with 5% skimmed milk in tris-buffered saline (TBS) 1X. Antibody against the RAGE (1/1000, AF1179, R&D Systems, Noyal-Châtillon-sur-Seiche, France), HMGB1 (1/10000, ab79823, Abcam), Myd88 (1/1000, ab133739, Abcam), and Diaphanous-1 (1/5000, ab1173, Abcam) were diluted in 5% skimmed milk-TBS 1X-TWEEN^®^ 20 0.1% and incubated overnight at 4°C. The next day, the membrane was washed three times with TBS 1X/TWEEN^®^ 20 0.1% and incubated at room temperature with a horseradish peroxidase coupled secondary antibody anti-goat or anti-rabbit (1/5000 or 1/10,000, respectively, BI 2403 or BI 2407, Abliance, Compiègne, France) for 1 h 30 min. The revelation was completed using an ECL clarity kit for Western blot on the ChemiDoc^TM^ imaging system (Bio-Rad). Image Lab Software (Bio-Rad) was used for quantification. Results are expressed as a mean of at least three independent experiments.

### Supernatant Protein Concentration

Before the Multiplex assays were completed, the supernatants of the treated explants were concentrated into 2 kDa centrifugal filter units (Vivacon^®^ 500, Sartorius, Aubagne, France) for protein concentration and purification, following the manufacturer’s instructions.

### Cytokine Multiplex Assay

The release of TNFα, IL1β, IL6, and IL8 in the culture media was tested using a MILLIPLEX MAP Human Cytokine/Chemokine Magnetic Bead Panel Milliplex^®^ MAP Kit (Merck Millipore, Molsheim, France) based on the Luminex^®^ xMAP^®^ technology, according to the manufacturer’s instructions (Biosource International). Finally, cytokine concentrations were normalized to total protein concentration, and the ratio “treated/untreated” was reported.

### Cellular Distress Determination

For the evaluation of the treatment impact on cell suffering, the release of the intracellular enzyme lactate dehydrogenase (LDH) into the cell media was quantified on a machine automate (Siemens Vista, Paris, France) using an enzymatic assay, following the manufacturer’s recommendations.

### Immunofluorescence

After permeabilization in PBS 1X/FBS 10%/Triton 0.1% over 1 h 30 min, the primary antibody against the RAGE (1/100, ab37647, Abcam) was applied on the FM sections overnight at 4°C. After three washes in permeabilization buffer, secondary antibody anti-rabbit Alexa Fluor 488 (1/1000, A21206, Life Technologies) was incubated for 2 h at room temperature. Slides were washed three times in TWEEN^®^ PBS 1X and incubated with Hoechst (15 min, dilution in PBS 1X 1/10,000; bisBenzimide H, 33258, Sigma-Aldrich). Finally, slides were mounted with CitiFluor^TM^ Tris-MWL 4–88 (Electron Microscopy Science) and examined under an Apotome Zeiss Imager microscope (magnification ×200). For negative controls, incubation without the primary antibody was performed.

### Statistical Analysis

The data expressed as mean ± standard error of the mean (*SEM*) are an average of duplicates or triplicates of at least three independent experiments. The comparison of means was performed by non-parametric test (Kruskal–Wallis one-way ANOVA test followed by a Dunn’s post-test for comparison of more than two conditions), or a Wilcoxon signed-rank test (comparing a fold change to one) using PRISM software 5.02 (GraphPad Software Inc.). For all studies, values were considered significantly different at *p* < 0.05(^∗^), *p* < 0.01(^∗∗^), and *p* < 0.001(^∗∗∗^).

## Results

### Are RAGE Axis Actors Expressed in Fetal Membranes During Pregnancy?

**FIGURE 1:**
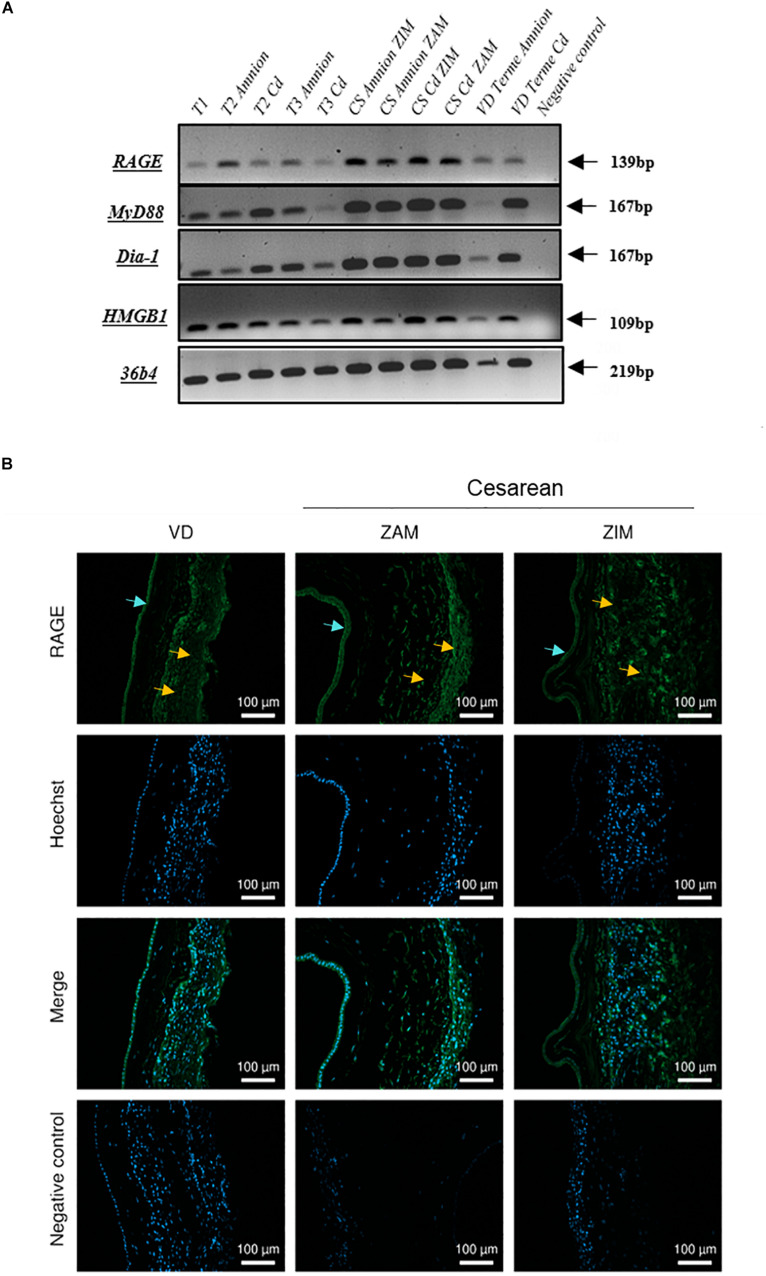
Expression of RAGE-signaling actors in human fetal membranes. **(A)** RNA expression of the RAGE, Myd88, Diaphanous-1, and HMGB1 was detected by RT-PCR on the amnion and choriodecidua (Cd) samples from the different trimesters (T1, T2, and T3), caesarean (CS), or vaginal (VD) delivery at term. Negative controls were performed in the absence of cDNA. **(B)** RAGE protein localization in human fetal membranes at term was investigated by immunofluorescence (green staining, Alexa488) on sections from vaginal delivery (VD) or caesarean (ZIM and ZAM) at magnification ×200. Cyan arrows indicate amniotic epithelium and yellow designate choriodecidua. Nuclei were counterstained with Hoechst (blue). Negative controls consisted of primary antibody-free incubation.

We investigated the mRNA expression profile of the RAGE, its adaptors and one ligand (HMGB1) in FMs on amnion and choriodecidua samples throughout pregnancy (first trimester: 1 to 13 weeks of gestation (WG); second trimester: 14–26 WG; third trimester: 27–37 WG; at term: 38–40 WG, by cesarean or vaginal delivery). RT-PCR experiments revealed that FMs expressed the RAGE, HMGB1, Myd88, and Diaphanous-1 in both layers, the amnion and choriodecidua in each of the stages considered (Figure 1A). No significant difference in RAGE expression was revealed by RT-qPCR between trimesters (data not shown). Plus, RAGE protein expression was also demonstrated in both layers obtained after vaginal delivery or caesarean section with the separate consideration of the ZIM and ZAM (Figure 1B).

### Is the RAGE Axis Actor Expression Layer- or Zone-Dependent at Term?

**FIGURE 2:**
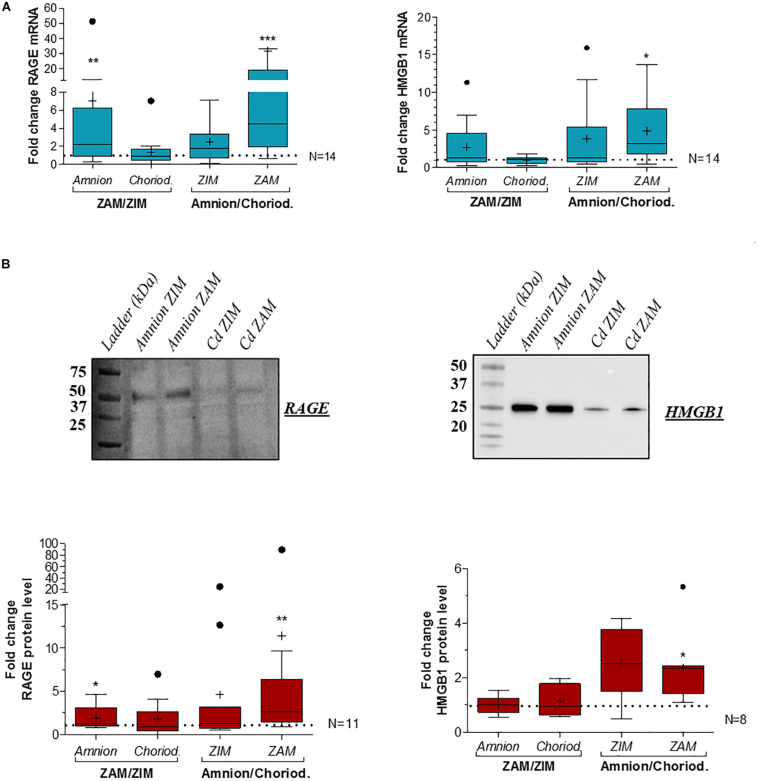
Quantification of RAGE and HMGB1 expression in human fetal membranes at term in the ZIM and ZAM. RAGE and HMGB1 expression were quantified by RT-qPCR (*N* = 14) **(A)** and Western blot (respectively, *N* = 11 and *N* = 8) **(B)** on the amnion and choriodecidua (Cd) at term in the ZIM and ZAM. To highlight an area (ZAM vs. ZIM) or tissue (Amnion vs. Choriodecidua) effect, ZAM is reported on ZIM (ZAM/ZIM in left panel) in both amnion and choriodecidua, and amnion is reported on choriodecidua (amnion/choriodecidua in right panel) in both ZIM and ZAM. Statistical fold change analysis was performed by a Wilcoxon signed-rank test comparing to one. * means *p* < 0.05, ** means *p* < 0.01, *** means *p* < 0.001. Results are presented in Tukey boxes, and means are indicated by “+.” Representative Western blot membranes indicate the band that was quantified proteins (RAGE: 50 kDa and HMGB1 at 25 kDa).

Based on RT-qPCR analysis at term, we revealed an overexpression of RAGE (Figure 2A, left panel) and HMGB1 (Figure 2A, right panel) in the amnion compared to the choriodecidua in consideration of the ZAM. Moreover, RAGE expression was also area-dependent: it was found to be significantly more expressed in the rupture zone (ZAM) than in the ZIM. These results were confirmed at the protein level for both using western blot analysis (Figure 2B, upper panel for representative experiment and Figure 2B, lower panel for quantification).

**FIGURE 3:**
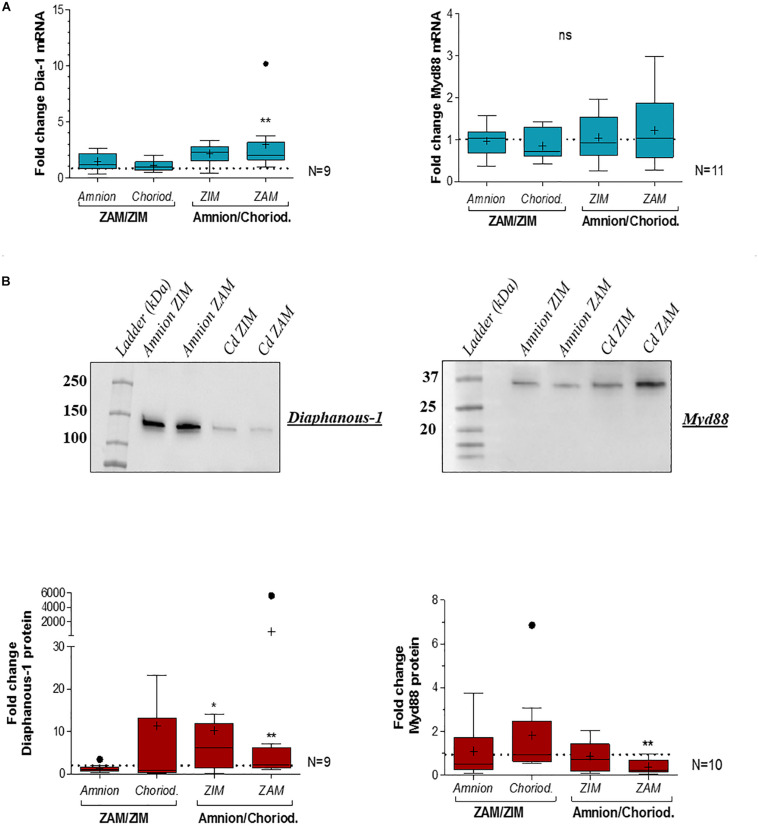
Quantification of RAGE-signaling adaptors in human fetal membranes at term in the ZIM and ZAM. Myd88 and Diaphanous-1 expressions were quantified by RT-qPCR (respectively, *N* = 11 and *N* = 9) **(A)** and Western blot (*N* = 9 and *N* = 10) **(B)** on the amnion and choriodecidua (Cd) at term in the ZIM and ZAM. To highlight an area (ZAM vs. ZIM) or tissue (amnion vs. choriodecidua) effect, ZAM is reported on ZIM (ZAM/ZIM in left panel) in both amnion and choriodecidua, and amnion is reported on choriodecidua (amnion/choriodecidua in right panel) in both ZIM and ZAM. Statistical fold change analysis was performed by a Wilcoxon signed-rank test comparing to one. * means *p* < 0.05, ** means *p* < 0.01. Results are presented in Tukey boxes, and means are indicated by “+.” Representative Western blot membranes indicate the band that was quantified (Diaphanous-1 was detected around 150 kDa and Myd88 at 37 kDa).

Furthermore, considering that the RAGE requires intracellular adaptor binding to induce a cellular response, we also investigated the expression of Diaphanous-1, Myd88, and TIR adaptator protein (TIRAP). First, TIRAP was found at very low levels for mRNA and not detected by immunoblot in both layers (data not shown). Furthermore, we revealed that Diaphanous-1 is overexpressed in the amnion for mRNA (only in ZAM) and protein level in both zones (Figures 3A,B, left panels). Plus, we found a Myd88 protein overexpression in the choriodecidua in comparison to the amnion in the ZAM (Figures 3A,B, right panels).

### Are the Amnion or Choriodecidua Able to Trigger an Inflammatory Response to Alarmins?

**FIGURE 4:**
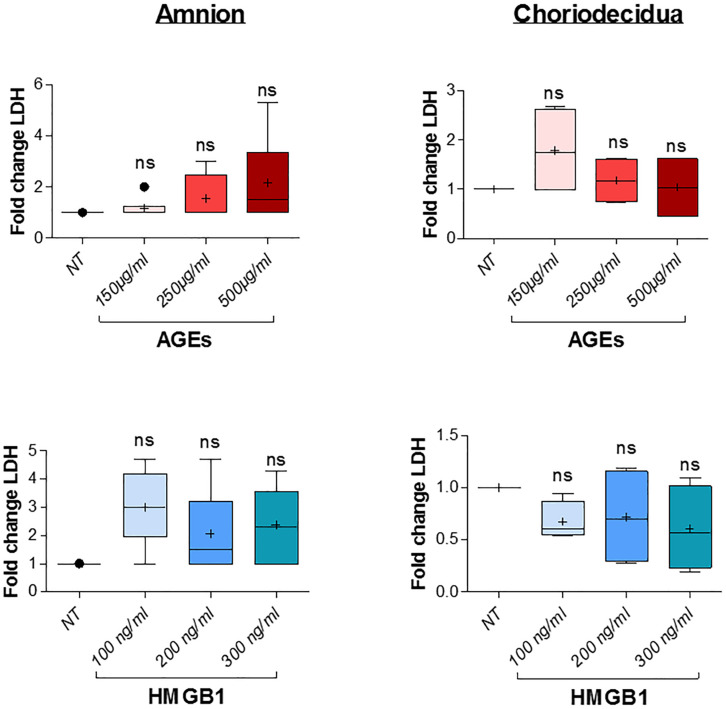
AGES or HMGB1 treatment effects on cell toxicity in the amnion and choriodecidua explants. Toxicity was evaluated by LDH release measurement in culture supernatants after 18 h of treatment with a dose effect of AGEs (150, 250, and 500 μg/ml) or HMGB1 (100, 200, and 300 ng/ml; *N* = 3 in duplicate). Statistical analysis was performed using a Kruskal–Wallis one-way ANOVA test followed by a Dunn’s post-test and showed no significant difference. Results are presented in Tukey boxes, and means are indicated by “+.”

**FIGURE 5:**
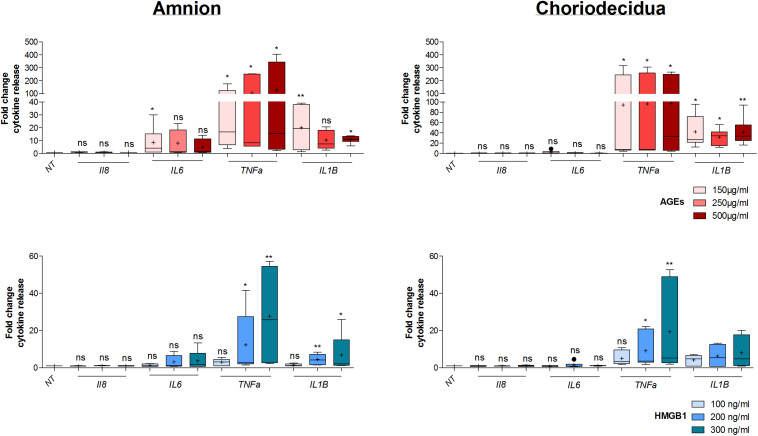
Dose response effect on cytokine release by AGEs and HMGB1 into fetal membrane explants. Evaluation of cytokine release (IL8, IL6, TNFα, IL1β) after 18 h of dose effect treatment of AGEs (150, 250, and 500 μg/ml) and HMGB1 (100, 200, and 300 ng/ml) on the amnion or choriodecidua explants was evaluated by Multiplex assay (*N* = 3 in duplicate). Statistical analysis was performed using a Kruskal–Wallis one-way ANOVA test followed by a Dunn’s post-test. * means *p* < 0.05, ** means *p* < 0.01. Results are presented in Tukey boxes, and means are indicated by “+.”

Before further investigation, cell toxicity that may have been caused by the induction of AGEs and HMGB1 treatments in the amnion and choriodecidua explants was checked. This was done using LDH release measurements in the culture media after 18 h of alarmin treatment and revealed no cell toxicity for each condition (three concentrations tested for each one; Figure 4). Then, we performed a dose response effect of AGEs and HMGB1 on cytokine release after 18 h of treatment on both the amnion and choriodecidua. First, in the amnion (Figure 5, upper left panel), we observed that AGEs did not stimulate IL8 release, but increased TNFα in the same way for all concentrations (150, 250, and 500 μg/ml) and IL1β (more at 150 than 500 μg/ml). Finally, we found that AGEs also induced IL6 release at 150 μg/ml. For the choriodecidua (Figure 5, upper right panel), the same responses as the amnion were found for IL8 and TNFα, and IL1β was increased regardless of the dose (more with 500 μg/ml). By contrast, induction was not relevant for IL6 at any concentration. A second time, we demonstrated that HMGB1 (Figure 5, lower panel) stimulated TNFα release in both tissues (at 200 and 300 ng/ml) and IL1β at the same doses but only in the amnion. Any significant induction could be reported by HMGB1 treatment for IL8 and IL6. Regarding our results, and in accordance with those already described in previous articles (Lappas et al., 2007; Plazyo et al., 2016), 500 μg/ml of AGEs and 200 ng/ml of HMGB1 were kept for the following SAGEs blocking experiments.

### Does Blocking the RAGE Modulate the Inflammatory Response Induced by Alarmins in Fetal Membranes?

**FIGURE 6:**
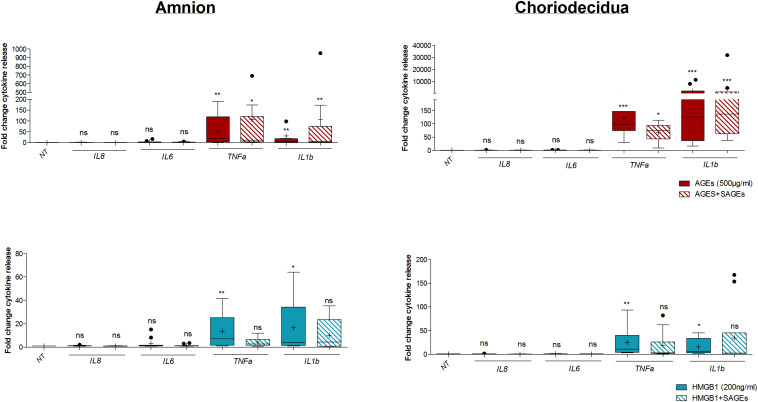
Impact of RAGE inhibition on the induction of inflammatory response by AGEs and HMGB1 alarmins in the amnion and choriodecidua. Pro-inflammatory cytokine (IL8, IL6, TNFα, IL1β) secretion was quantified by Multiplex assay after 18 h of treatment with AGEs (500 μg/ml) or HMGB1 (200 ng/ml) in the presence or absence of RAGE inhibitor, SAGEs (500 μg/ml) (*N* = 5 in duplicate). Each treated condition was reported to corresponding not treated (NT) either without SAGEs or with SAGEs, which were both fixed to one. Statistical analysis was performed using a Kruskal–Wallis one-way ANOVA test followed by a Dunn’s post-test. * means *p* < 0.05, ** means *p* < 0.01, *** means *p* < 0.001. Results are presented in Tukey boxes, and means are indicated by “+.”

Finally, to investigate whether AGEs and HMGB1 alarmins induce a RAGE-dependent inflammatory response, we measured pro-inflammatory cytokine release in the amnion and choriodecidua co-treated with or without SAGEs (a RAGE inhibitor) for 18 h. Results demonstrated a significant lower TNFα release induction by AGEs (Figure 6, upper panel) and HMGB1 (Figure 6, lower panel) when the RAGE was inhibited by SAGEs in the amnion and choriodecidua. Plus, we noticed that AGEs and HMGB1 stimulated IL1β secretion in both layers, but in the presence of SAGEs, this induction was not any more significant for HMGB1. Finally, we found no impact on either IL8 or IL6.

## Discussion

Since these last years, more and more studies have underlined the importance of sterile inflammation in the weakening of FMs as a key event of the ROM (hopefully after 37 weeks of gestation). It is now considered that AF is a source of specific molecules called alarmins (or DAMPs), which are endogenously expressed and produced when cells are suffering. For example, HMGB1 is normally a nuclear protein implicated in DNA reparation, but when cells are in danger, HMGB1 is released and becomes an alarmin, triggering an inflammatory cascade. As another kind of DAMPs, AGEs are formed by the non-enzymatic Maillard reaction, between sugars and proteins, lipids, or nucleic acids (John and Lamb, 1993), and many inflammatory diseases are linked to an accumulation of these AGEs in tissues (Kang et al., 2012; Guedes-Martins et al., 2013; Wautier et al., 2014). Above all, both AGEs and HMGB1 have been described as activating inflammatory response in gestational tissues (placenta, FMs, umbilical cord) and were found to be increased in cases of pPROM. Indeed, HMGB1 has been found to be more elevated in the AF due to a release caused by damaged FMs occurring during intra-amniotic inflammation found during preterm birth (Bredeson et al., 2014; Baumbusch et al., 2016). This could be an exacerbation of inflammatory processes mediated by HMGB1, a major player in labor events (Stephen et al., 2015). In addition, AGEs levels in maternal plasma were described as more important during the first trimester for pregnancies with preterm labor or pPROM (Kansu-Celik et al., 2019). However, there is still a lack of knowledge about which receptor recognizes these alarmins and causes inflammation in the FMs. Some studies have described an overexpression of the RAGE in the placenta and maternal serum in cases of pPROM and also a progressive increase of the soluble isoform of RAGE (sRAGE), acting as a decoy, during pregnancy and then finally decreasing at term (Romero et al., 2008; Yan et al., 2018). Moreover, plasmatic sRAGE levels were found to be lower in patients with pPROM, suggesting an over-activation of the RAGE pathway (Hájek et al., 2008). In this way, our work aimed to enlighten the implication of the RAGE in sterile inflammation in FMs.

The expression of the RAGE in FMs has already been described at term but had not been described during the different trimesters of pregnancy. Presently, we conducted a global exploration of the RAGE axis in both FMs layers (amnion and choriodecidua). First, we proved that FMs not only expressed the RAGE and HMGB1 during all three trimesters of pregnancy, but it also expressed Diaphanous-1 and Myd88, two intracellular adaptors required for inflammatory activity of the RAGE. After that, we observed the presence of the RAGE protein in the amniotic epithelium, the layer directly exposed to AF alarmins and also in all choriodecidua. Thus, we found a differential RNA and protein expression between not only the amnion and choriodecidua and also between the ZAM and the rest of the FMs, the ZIM. Indeed, we showed the overexpression of the RAGE in the amnion compared to the choriodecidua and, above all, in the ZAM compared to the ZIM. These findings strengthen the idea of RAGE participation and importance in the ROM process as previously suggested (Rzepka et al., 2015). Plus, we demonstrated HMGB1 levels to be more important in the amnion. This was not very surprising; indeed, literature already described an increase in sterile inflammation linked with HMGB1 on the fetal side of the FMs, more precisely, in the amnion epithelial cells (Romero et al., 2011). But our work brings the first data on RAGE adaptors in fetal membranes and this is not negligible. In fact, it is currently well known that the RAGE is deficient in intrinsic tyrosine kinase activity and requires intracellular adaptors to induce cell signaling cascades. In this way, a yeast-two-hybrid experiment was achieved and identified a binding partner of RAGE cytosolic domain, the protein Diaphanous-1. Meanwhile, there is no proof that Diaphanous-1 is required for all RAGE induced-transduction cascades; however, some studies reported its implication in protein/signal pathway stimulation triggered by RAGE ligands (Xu et al., 2010; Touré et al., 2012). Hudson et al. (2008) also demonstrated that downregulation of Diaphanous-1 expression by RNA interference inhibited RAGE-mediated activation of Rac-1 and Cdc42 and, in parallel, RAGE ligand-stimulated inflammatory, vascular, and cell migration responses. Diaphanous-1 aside, the RAGE owns other adaptor proteins, such as TIRAP or MyD88, shared with the toll-like receptors TLR2 and TLR4. The Sakaguchi group revealed that ligand binding leads to phosphorylation of the RAGE cytoplasmic domain by protein kinase Cζ (pSer391), promoting TIRAP and MyD88 interaction. Furthermore, blocking TIRAP and MyD88 considerably abolished ligand-activated RAGE inflammatory signaling (Akt, p38 MAP kinase, NFκB) (Sakaguchi et al., 2011). In our study, we demonstrated an overexpression of Diaphanous-1 in the amnion, unlike Myd88, which was found to be expressed more in the choriodecidua. These data may suggest a layer-specific signaling couple, RAGE/Diaphanous-1 in the amnion and RAGE/Myd88 in the choriodecidua. Additionally, considering that RAGE and TLR2/4 partly share an intracellular signaling pathway, including MyD88 binding, we could suppose cooperation between RAGE and TLRs in immune response in choriodecidua, explaining why MyD88 was overexpressed in this layer, closer to the genito-urinary microbiota. Indeed, fetal membranes are already known to respond to different types of bacteria by modifications of TLR expression patterns (Abrahams et al., 2013).

As previously stated, AGEs or HMGB1 have been described as inducing cytokine release (IL6, IL8, TNFα, IL1β) in the FMs but without making a distinction between both layers. To investigate a possible differential response to ligands between the amnion and choriodecidua, we decided to perform our treatments with a dissociation of these two sheets using either AGEs or HMGB1. First, we confirmed results from previous studies with TNFα and IL1β release in response to AGEs and HMGB1 in both the amnion and choriodecidua (Lappas et al., 2007; Bredeson et al., 2014; Plazyo et al., 2016). In addition to such results, we did not find the same results for IL8 and IL6 with their release not being stimulated by any dose except for IL6 in the amnion by AGEs at 150 μg/ml. This contrast with previous results can be explained by the use of different concentrations of alarmins. For example, the Lappas group used concentrations of 1 mg/ml of AGEs, and those used for HMGB1 were between 10 ng and 50 μg/ml for Plazyo and colleagues and 1 and 50 ng/ml by the Bredeson team. Then, in our study, for IL6 release, we dissociated layers and could, therefore, hypothesize that only the amnion produces such interleukin in response to AGEs or to HMGB1.

Finally, the major finding of this study was that RAGE inhibition by SAGEs decreased or aborted the TNFα release for both alarmins, indicating that RAGE is required for FMs to initiate this cytokine production. However, contrary to choriodecidua, in amnion, it seems RAGE is not the only actor needed in TNFα release induction because the induction was diminished and not aborted. Globally, this result is of primary importance, as TNFα could activate NFκB complex and the production of the granulocyte macrophage colony-stimulating factor (GM-CSF), which is described to be the critical intermediate for FMs weakening by the intervention of specific proteases (Kumar et al., 2014). The release of IL1β was only inhibited by SAGEs when tissues were treated by HMGB1 and not by AGEs. Considered together, the results obtained in our work proved for the first time that the RAGE is directly implied in the inflammatory response in human FMs in a ligand and layer-dependent manner.

Our study constitutes the direct evidence of RAGE action in FMs weakening, which is an essential process for the ROM, adding a feature of the pathophysiological partition of the RAGE in the story of childbirth. Further studies are required to elucidate which intracellular pathway, among NFκB and MAPK kinases, for example, leads to this RAGE-dependent cytokine release in FMs, but also which cellular type inside the amnion or choriodecidua has the ability to react to the presence of alarmins.

## Data Availability Statement

All datasets generated for this study are included in the article/supplementary material.

## Ethics Statement

The Institutional Local Ethics Committee, structure of the University Hospital of Clermont-Ferrand (specialized for Human clinical questions) approved this study and the research protocol. Healthy fetal membranes were collected after receiving oral informed consent (according to the French law named “Huriet-n°88–1138” which considers placenta and fetal membranes as chirurgical wastes) from the patients in the “Centre Hospitalier Universitaire Estaing” (Clermont-Ferrand, France).

## Author Contributions

HC designed and performed the experiments, and wrote the manuscript. ML and CB helped HC to carry out some experiments. JD helped with Multiplex assays. RM-Q performed lactate dehydrogenase assays. DG allowed HC to obtain human fetal membranes from patients in the Centre Hospitalier Universitaire Estaing (Clermont-Ferrand, France). VS and LB supervised the project.

## Conflict of Interest

The authors declare that the research was conducted in the absence of any commercial or financial relationships that could be construed as a potential conflict of interest.
